# Proliferation of osteoblast precursor cells on the surface of TiO_2_ nanowires anodically grown on a β-type biomedical titanium alloy

**DOI:** 10.1038/s41598-022-11981-4

**Published:** 2022-05-12

**Authors:** Leonardo Fanton, Frida Loria, Mario Amores, M. Ruth Pazos, Cristina Adán, Rafael A. García-Muñoz, Javier Marugán

**Affiliations:** 1 School of Mechanical Engineering, University of Campinas (Unicamp), Rua Mendeleyev, 200, Campinas, São Paulo, 13083-860 Brazil; 2 Department of Chemical and Environmental Technology, Universidad Rey Juan Carlos, C/ Tulipán S/N, Móstoles, 28933 Madrid, Spain; 3 Laboratorio de Apoyo a la Investigación, Hospital Universitario Fundación Alcorcón, C/ Budapest 1, Alcorcón, 28922 Madrid, Spain

**Keywords:** Chemical engineering, Mechanical engineering

## Abstract

Studies have shown that anodically grown TiO_2_ nanotubes (TNTs) exhibit excellent biocompatibility. However, TiO_2_ nanowires (TNWs) have received less attention. The objective of this study was to investigate the proliferation of osteoblast precursor cells on the surfaces of TNWs grown by electrochemical anodization of a Ti-35Nb-7Zr-5Ta (TNZT) alloy. TNT and flat TNZT surfaces were used as control samples. MC3T3-E1 cells were cultured on the surfaces of the samples for up to 5 days, and cell viability and proliferation were investigated using fluorescence microscopy, colorimetric assay, and scanning electron microscopy. The results showed lower cell proliferation rates on the TNW surface compared to control samples without significant differences in cell survival among experimental conditions. Contact angles measurements showed a good level of hydrophilicity for the TNWs, however, their relatively thin diameter and their high density may have affected cell proliferation. Although more research is necessary to understand all the parameters affecting biocompatibility, these TiO_2_ nanostructures may represent promising tools for the treatment of bone defects and regeneration of bone tissue, among other applications.

## Introduction

Titanium and its alloys have a high specific strength (strength-to-weight ratio) and the best biocompatibility among metals. Titanium naturally forms an oxide (TiO_2_) on its surface, which effectively protects it from corrosion, even in aqueous media. Therefore, despite its relatively high production costs, titanium is advantageous for many applications, particularly in the aerospace^1^ and biomedical industries^2^. At low temperatures, pure titanium has a hexagonal close-packed crystal structure, known as the α phase, which undergoes an allotropic transformation at 882 °C to a body-centered cubic structure, known as the β phase. To stabilize the β phase at lower temperatures, alloying elements such as Mo, Nb, V, and Ta can be added to titanium. Titanium alloys are widely used to manufacture biomedical materials, particularly those used to replace hard tissues. The β-phase of titanium exhibits a considerably low elastic modulus, which increases the mechanical compatibility between the implant and bone. The elastic modulus of an implant should be as close as possible to that of bone to reduce the stress-shielding effect^3^, which is a serious issue that can cause bone mass loss (osteopenia) and eventually lead to implant failure. The elastic moduli of commonly used biomaterials such as commercially pure titanium or stainless steel may be up to six times higher than that of bone^4^. In recent years, β-type titanium alloys, based on the quaternary Ti-Nb-Zr-Ta system, have been studied for surgical implant applications^5^ because of their superior biocompatibility and low elastic moduli. One such material is Ti-35Nb-7Zr-5Ta (TNZT), a metastable β-titanium alloy with a low elastic modulus (approximately 60 GPa^6,7^) that is free of toxic elements. Another critical concern is the ability of the implant to osseointegrate, that is, to form a stable fixation with the bone. When an implant is inserted into the human body, it generates an inflammatory response, which ends with implant encapsulation by collagen molecules. This capsule formation is difficult to avoid, but titanium-based materials show minimal encapsulation compared to other biomedical metals such as stainless steel and Co-Cr alloys^2^.

Despite the advantages of titanium over other metallic biomaterials, further advances are necessary to enhance osseointegration and reduce the implant rejection rate. As implant biocompatibility is closely related to its surface chemistry and topography, surface modifications of titanium have been extensively studied^8,9^, including the growth of TiO_2_ nanotubes (TNTs)^10^ by electrochemical anodization. The latter involves the application of an electric potential between the titanium or titanium alloy substrate (anode) and a counter electrode (cathode), separated by a fluoride-containing electrolyte. The formation of TNTs during anodization is due to a combination of simultaneous processes, which can be summarized as a competition between the field-assisted growth of the TiO_2_ layer and the chemical dissolution of TiO_2_ by the fluoride-containing electrolyte, preferentially occurring at the tube base^11^. During the anodic growth of TNTs, TiO_2_ nanowires (TNWs) can be formed on the upper portion of TNTs by a process of vertical division of TNTs, known as the “bamboo-splitting model”^12^. The final nanostructure is composed of TNTs with TNWs on top, and the length of TNWs can be even longer than that of TNTs. The anodization parameters required for the formation of TNWs may vary depending on the substrate (anode) composition, and the TNZT alloy favors their formation^13^. TNWs can also be synthesized by other techniques, such as electrospinning, laser ablation, and oxidation^14^. The term TiO_2_ nanofibers (TNFs) is also used in the literature to describe structures similar to TNWs. Although the difference between these two terms is not clear, TNWs typically have diameters on the order of tens of nanometers, whereas TNFs have larger diameters of up to 1 μm^15^. Both TNWs and TNFs have a high surface area, which may help cells attach and proliferate. Studies on the biocompatibility of polymeric fibrous scaffolds^16^ have shown that this type of morphology offers a favorable environment for cells, owing to its similarity to the native bone extracellular matrix. In addition, anodically grown TNWs have the advantage that they can be easily grown even on implants with complex geometries and, as they grow directly from the substrate, no additional step is needed to attach them to the implant surface.

A significant number of studies^17^ have shown that TNTs are promising biomedical materials that provide good support for cell attachment and proliferation. However, the biocompatibility of TNW (or TNF) coatings has received little attention in the literature; what studies there are have had many differences in methods, designs and results, and are therefore difficult to compare^18–22^. These studies are summarized in Table 1. In addition, most studies on the fabrication and application of these TiO_2_ nanostructrures have employed CP-Ti or Ti-6Al-4V, which are the most traditional and commonly used alloys. Meanwhile, more attention should be paid to low-modulus β-type titanium alloys, such as TNZT, designed for biomedical applications. In addition to its enhanced mechanical compatibility with the bone, the TNZT alloy is advantageous over CP-Ti for the anodic growth of TiO_2_ nanostructures, producing threefold longer TNTs and showing the much easier formation of TNWs^13^. To the best of our knowledge, no study has explored the cellular behavior on the surface of TNWs that were anodically grown on biomedical β-type titanium alloys. Therefore, this study aimed to investigate the biocompatibility of TNWs anodically grown on the TNZT alloy by cultivating osteoblastic precursor cells on their surfaces. TNT coated and flat TNZT surfaces were used as control samples.

**Table 1:** Studies of bone cell behavior on surfaces covered with TiO_2_ nanowires/nanofibers.

Author	Method	Substrate	Morphology	Nanowire/nanofiber diameter	Cell line	Cell proliferation
Fanton et al. (this study)	Anodization	Ti-35Nb-7Zr-5Ta	Nanowires	28 nm	MC3T3-E1	**Lower** than that on the flat TNZT and TNT surfaces
Chen et al.^18^	Electrospinning	Pure Ti	Nanofibers	130, 200, and 320 nm	MG-63	**Higher** (by ~ 20%) on the nanofibers of 200 nm in diameter than that on flat Ti after 6 days in culture. Similar for the other diameters
Wang et al.^19^	Electrospinning	No substrate	Nanofiber meshes	184 and 343 nm	MG-63	**Lower** than that on a polystyrene plate. No difference between the nanofibers with the two different diameters
Dinan et al.^20^	Thermal oxidation	Ti-6Al-4 V	Nanowires (predominantly) and nanoribbons	~ 500 nm	Human osteosarcoma	**Higher** (by ~ 40%) than that on the flat Ti-6Al-4 V alloy after 15 h of culture
Huang et al.^21^	Atomic layer deposition of TiO_2_ to cover Si nanofibers	Ti-6Al-4 V	Nanofibers with a bird’s nest morphology	60 nm	MC3T3-E1	**Lower** than that of for Si, SiO_2_ nanofibers, and flat Ti-6Al-4 V
Chang et al.^22^	Rotating anodization	Pure Ti	Nanowires	Not measured	MG-63	**Higher** (by ~ 100%) than that on flat Ti after 21 days of culture

## Experimental

### Alloy production

The Ti-35Nb-7Zr-5Ta (wt.%) alloy was produced by voltaic arc melting of pure elements on a water-cooled copper crucible under an Ar atmosphere. The ingot was remelted at least 10 times and flipped on the crucible after each time to ensure homogeneity. Subsequently, it was encapsulated in a quartz glass tube filled with Ar and homogenized at 1000 °C for 24 h to eliminate element microsegregation. The ingot was then cold-rolled in multiple passes to reduce the thickness by approximately 75%, resulting in a plate with a thickness of 2 mm. The plate was subsequently annealed under an Ar atmosphere at 800 °C (above the β-transus temperature) for 1 h, followed by water quenching.

### TNW synthesis

The TNZT alloy plate was cut into pieces of approximately 15 × 15 mm for the synthesis of TNWs by electrochemical anodization. The surfaces of the samples were prepared by sanding with abrasive papers up to 1200 grit and chemically polished for 10 s in an acid solution composed of HF and HNO_3_ (1:1). Anodization was performed in an electrolytic cell with a volume capacity of approximately 100 ml (50 mm in diameter and 50 mm in height) using a platinum mesh of 30 × 30 mm as the cathode. The TNZT sample, which was the anode of the system, was in contact with the electrolyte solution through a round window of 8 mm in diameter located at half of the cell height. The anode and cathode were connected to a power supply (Elektro-Automatik 8200–70, Viersen, Germany) operating in continuous voltage mode. The electrolyte was stirred continuously during anodization using a magnetic stir bar. The anodization parameters used were based on a previous study^13^. To grow TNWs, an organic electrolyte containing ethylene glycol with 0.5 wt.% of NH_4_F and 10 vol.% of water was used, and a voltage of 20 V was applied for 12 h. Immediately after anodization, samples were rinsed with deionized water.

### Control samples

TNT coated and flat TNZT samples were used as control surfaces. Despite TNTs and TNWs present very different morphologies, both can be synthesized by a similar anodization process, and the obtention of one or another depends only on the parameters used. TNT samples were chosen as control because TNTs already have been extensively studied and they are known to present excellent biocompatibility^10^. Considering that TNTs are easier and faster to be obtained^13^, the use of anodized TNWs as a biomaterial would be only justified if their biocompatibility were superior or if they presented any other advantage over the TNTs.

The TNTs were synthesized with a similar procedure used for the growth of TNWs, except different anodization parameters were chosen. Instead of the organic electrolyte, an aqueous electrolyte containing 0.3 vol.% of HF was used, and a voltage of 20 V was applied for 1 h. The aqueous electrolyte limits the thickness of the TiO_2_ layer and prevents the formation of TNWs^13^. The flat samples were produced by sanding the TNZT plates with abrasive paper up to 1200 grit and chemically polishing them for 10 s in an acid solution composed of HF and HNO_3_ (1:1).

### Wettability tests

The wettability of the TNW, TNT, and flat TNZT surfaces was evaluated by measuring the contact angle between a drop of water and the sample surface, following the guidelines described in the ASTM D7334-08 standard^23^. Three different drying procedures were employed before measuring the contact angle: (i) drying with N_2_ flow for about two minutes, (ii) drying under vacuum, and (iii) immersion in deionized water for 24 h followed by vacuum drying. A micropipette fixed in a vertical position was used to gently deposit 5 μl of deionized water on the sample surface. The shape of the water drop was recorded using a portable digital microscope camera. The contact angle between the water drop and the surface was measured using the contact angle plugin for the ImageJ software^24^ and at least three samples for each condition.

### Cell culture

The MC3T3-E1 osteoblast precursor cell line was obtained from Sigma–Aldrich (ECACC, Cat. No. 99072810) and maintained in alpha minimum essential medium (α-MEM; Pan Biotech) supplemented with 10% fetal bovine serum (Sigma–Aldrich) and penicillin/streptomycin (Dominique Dutcher) at 37 °C and 5% CO_2_, with the medium changed every 2 days. When cells became confluent, they were detached and passaged using 0.25% trypsin (Dominique Dutcher). Before cell culture, all samples were sterilized with ethanol (70%) washing for 1 h, followed by water washing and ultraviolet (UV) radiation for 20 min. The exposure of the TiO_2_ surfaces to UV could in principle alter their wettability, but this change is reversed when samples are immersed in the aqueous environment during cell culture. For biocompatibility experiments, 80 µl of complete α-MEM containing 6000 cells was placed on top of the flat TNZT, TNT, and TNW surfaces. After 3 h of incubation, 3 ml of the medium was added to the Petri dishes (35 mm) containing the samples.

### Cell viability

#### Calcein assay

Cell proliferation was estimated by measuring calcein acetoxymethyl ester uptake every 24 h. In living cells, the nonfluorescent calcein was converted into green fluorescent calcein after hydrolysis of acetoxymethyl ester by intracellular esterases. Briefly, cells were incubated at 37 °C for 30 min with 3 μM calcein AM (Molecular Probes, Life Technologies), then washed with HBSS (Gibco), and visualized under a Nikon Eclipse 90i fluorescence microscope. At least five randomly selected fields were captured and analyzed per experimental condition using a Nikon DXM 1200F camera. To estimate the number of calcein-positive cells, total fluorescent pixels were counted per field, with a previously adjusted background for all images. The ImageJ software version 1.51j8 (National Institutes of Health, MD, USA) was used for the analysis.

#### MTT assay

For the MTT (3-(4,5-dimethylthiazol-2-yl)-2,5-diphenyltetrazolium bromide) assay, cells were incubated at 37 °C with 0.5 mg/ml MTT reagent (Sigma–Aldrich). After 2 h, 2 ml of 2-propanol was added to each culture plate, and the absorbance was measured at 560 nm using a microplate reader (Thermo Multiskan Ex). MTT measurements were performed 1, 3, and 5 days after plating cells.

#### Statistical analysis

Results are expressed as the mean ± SEM, and the data were analyzed using the GraphPad Prism software version 7.04 (GraphPad Software, San Diego, CA, USA). Calcein-positive pixels and MTT analysis were compared among the groups over time using a two-way analysis of variance, followed by Dunnett's post-hoc test.

### Scanning electron microscopy (SEM)

Cell morphology was observed by SEM (Hitachi S-3000N) with an accelerating voltage from 0.3 to 30 kV. Before characterization by SEM, cells were cultured on the surfaces of the TNW and control samples for 48 h. Subsequently, the cells were fixed, dehydrated, dried, and coated with gold. The fixation process started with four consecutive washes with cacodylate buffer (pH 7.4) for 15 min, fixation with 2% glutaraldehyde in cacodylate buffer for 1 h, and fixation with 1% osmium tetroxide in cacodylate buffer for 2.5 h. After five washes with distilled H_2_O, the samples were incubated with 1% tannin in the dark for 1 h for dehydration. The samples were then dehydrated with increasing concentrations of ethanol (70%, 80%, 90%, 96%, and 100%) for 10 min each and dried using three solutions of hexamethyldisilazane (HMDS) in the following proportions: one part of HMDS + two parts of 100% alcohol (20 min); equal parts of HMDS and 100% alcohol (20 min); and three parts of HMDS + one part of 100% alcohol (20 min). Finally, the three samples were coated with a thin layer of Au using a sputter metallization Q150T-S system (Quorum Technologies).

### X-ray photoelectron spectroscopy and X-ray diffraction

X-ray photoelectron spectroscopy (XPS) analysis was performed with a Physical Electronics (PHI Versa Probe II Scanning XPS Microprobe) spectrometer using monochromatic radiation Al Kα (1486.6 eV, 100 μm, 100 W, 20 kV) as the excitation source and a dual-beam charge neutralizer. The high-resolution spectra were acquired with a pass energy of 29.35 eV and an X-ray beam diameter of 100 mm. The NIST Standard Reference Database^25^ was used to index the XPS spectra. X-ray diffraction (XRD) analysis (2q scans) was carried out with a Panalytical X’Pert Pro diffractometer using Cu-ka radiation (wavelength = 1.5406 Å).

## Results and discussion

**Figure 1:**
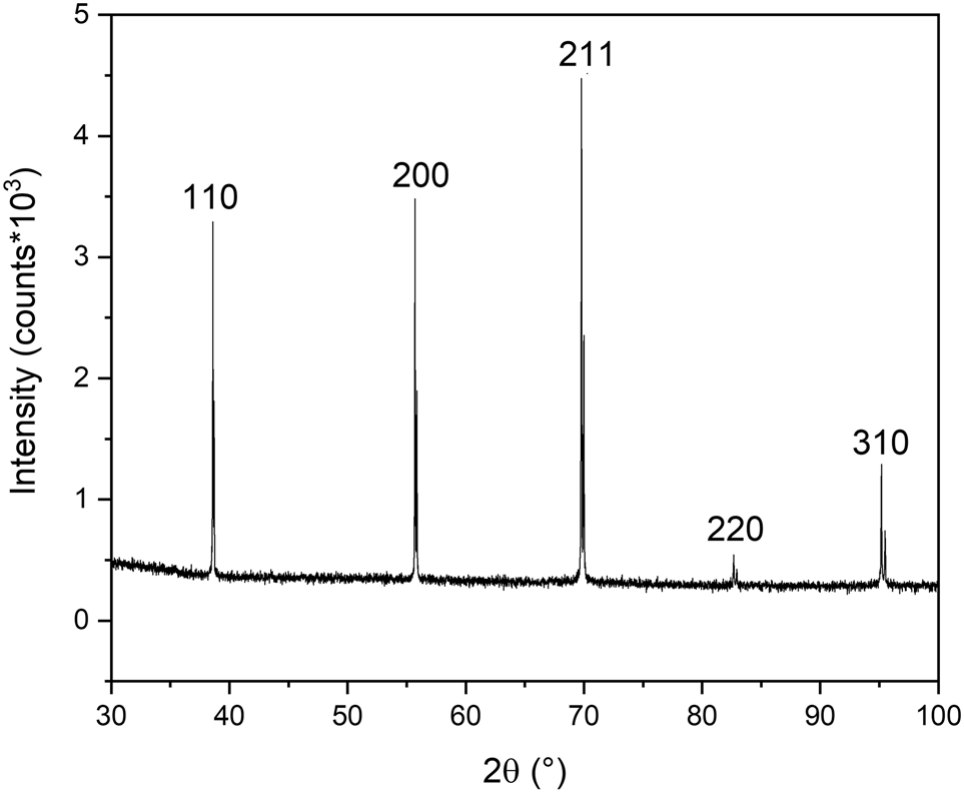
XRD analysis of the TNZT alloy used as the substrate for the anodic growth of TNTs and TNWs. All the reflections observed are from the body-centered cubic (β) phase of titanium.

XRD analysis of the TNZT alloy used as the substrate for the anodic growth of TNTs and TNWs was carried out to confirm whether the samples have the expected phase composition. Figure 1 shows the obtained pattern. All the reflections seen in the figure are from the body-centered cubic (β) phase of titanium (Powder Diffraction File database—PDF number 01-071-9955), as expected for this alloy.

**Figure 2:**
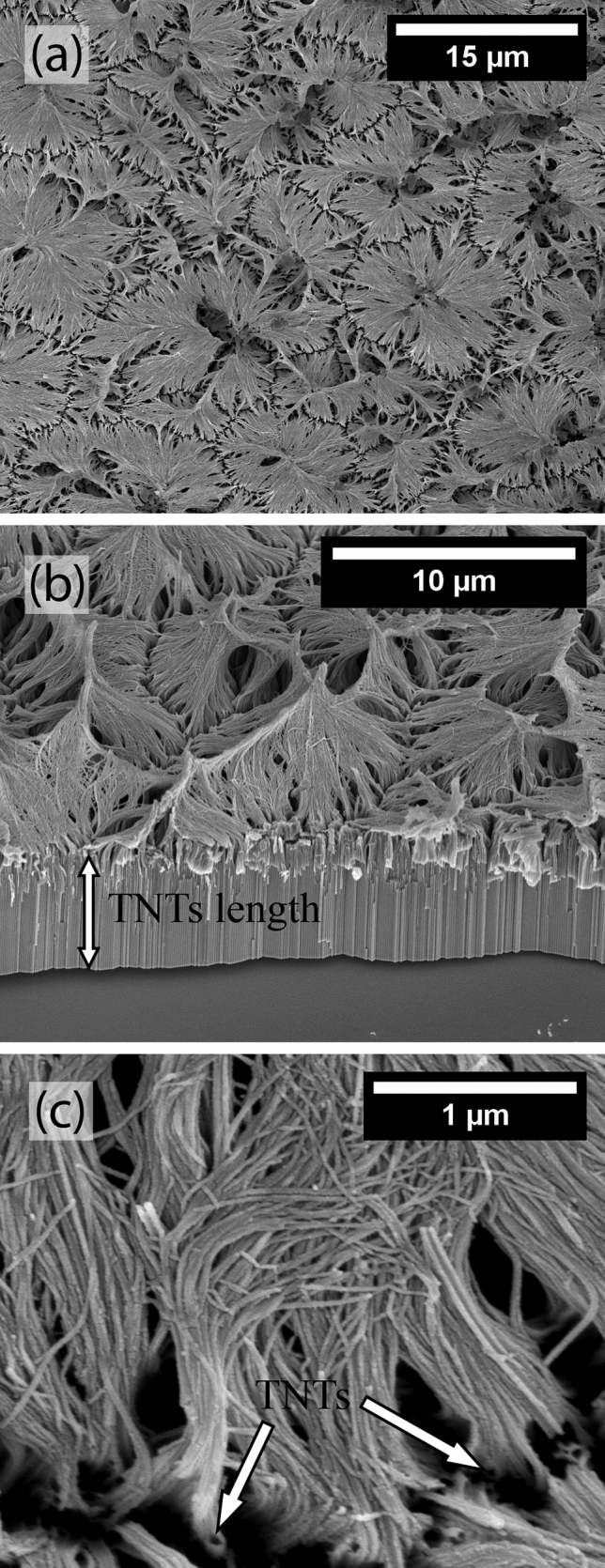
FEG-SEM micrographs of a TNZT sample anodized in the organic electrolyte at 20 V for 12 h, which resulted in the formation of TNWs on top. (**a**) Top-view image, (**b**) angled-view image, and (**c**) top-view image at a higher magnification.

Anodization of the TNZT samples in the organic electrolyte was carried out at 20 V for 12 h and resulted in the entire surface being densely covered with TNWs, as shown in Fig. 2a. The TNWs appeared to be very flexible and were grouped in clusters, as also observed in a previous study^13^. The TNWs grew on top of TNTs, as observed in Fig. 2b, in accordance with the bamboo-splitting model proposed by Lim and Choi^12^. The TNTs were approximately 4.6 μm in length and 80 nm in diameter. Figure 2c presents a higher-magnification image of the TNWs, showing their growth from the TNT walls. The precise length of the TNWs was difficult to measure because of their tangled morphology, but they seemed to be equal in length to the TNTs. The average diameter of the nanowires was approximately 28 nm.

**Figure 3:**
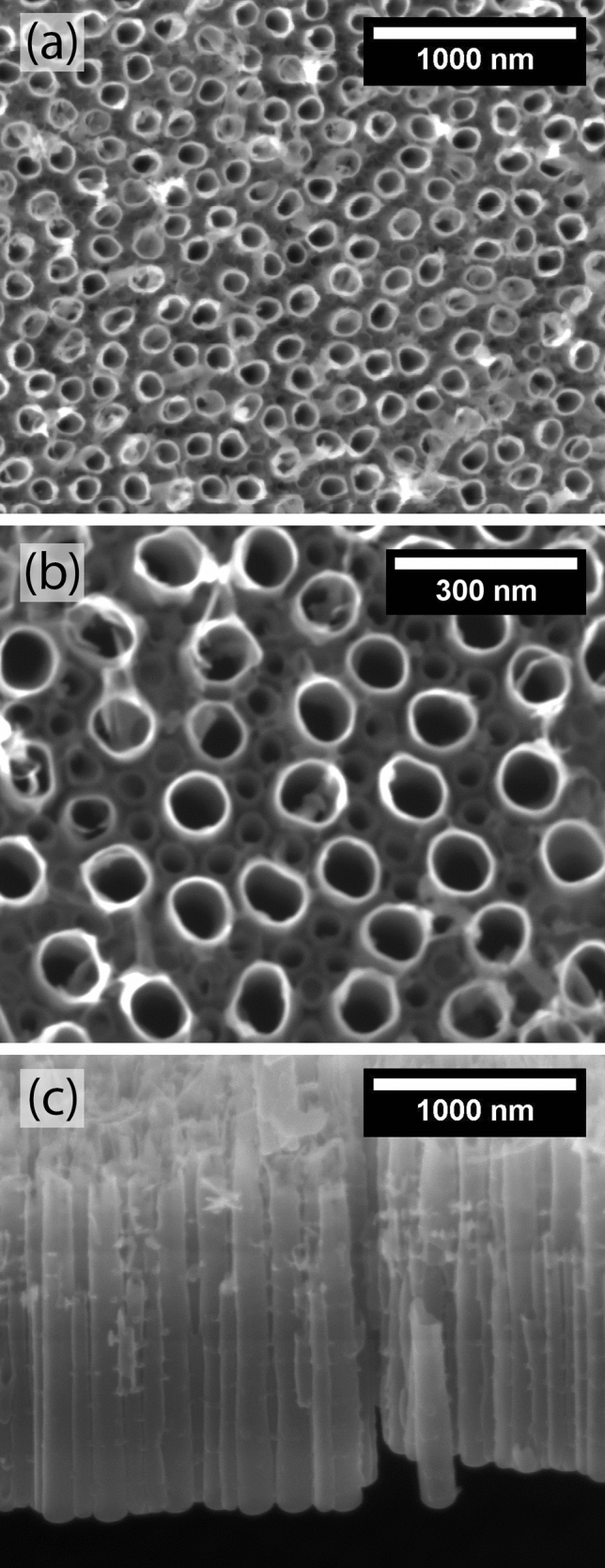
FEG-SEM micrographs of a TNZT sample anodized in the aqueous electrolyte at 20 V for 1 h. (**a**,**b**) Top-view and (**c**) side-view images.

The TNT-coated samples were obtained by anodization of the TNZT alloy with the aqueous electrolyte at 20 V for 1 h. TNTs with a relatively uniform morphology were generated, with thin walls and well-opened mouths (Fig. 3a). The nanotubes formed under this anodization condition could be divided into two groups based on their sizes, as observed in an earlier study^13^. The nanotubes in the first group were longer and wider, with an average length of 1.65 μm and a diameter of approximately 109 nm. The nanotubes from the second group surrounded those from the first group (Fig. 3b) and had a length of approximately 1.1 μm and a diameter of 76 nm. The difference in length between the two groups was better visualized in a side-view image (Fig. 3c).

Table 2 provides an overview of the anodization parameters used and the resulting morphologies.

**Table 2:** Morphology of TiO_2_ nanostructures obtained after anodization using different parameters.

TiO_2_ nanostructure	Anodization parameters		TNT dimensions		TNW diameter
	Electrolyte	Voltage/time	Length	Diameter	
TNWs	Organic	20 V, 12 h	4.7 ± 0.3 μm	80 ± 3 nm	28 ± 3 nm
TNTs (control)	Aqueous	20 V, 1 h	Group I: 1.70 ± 0.05 μm Group II: 1.10 ± 0.04 μm	Group I: 109 ± 6 nm Group II: 76 ± 3 nm	No TNWs formed

**Figure 4:**
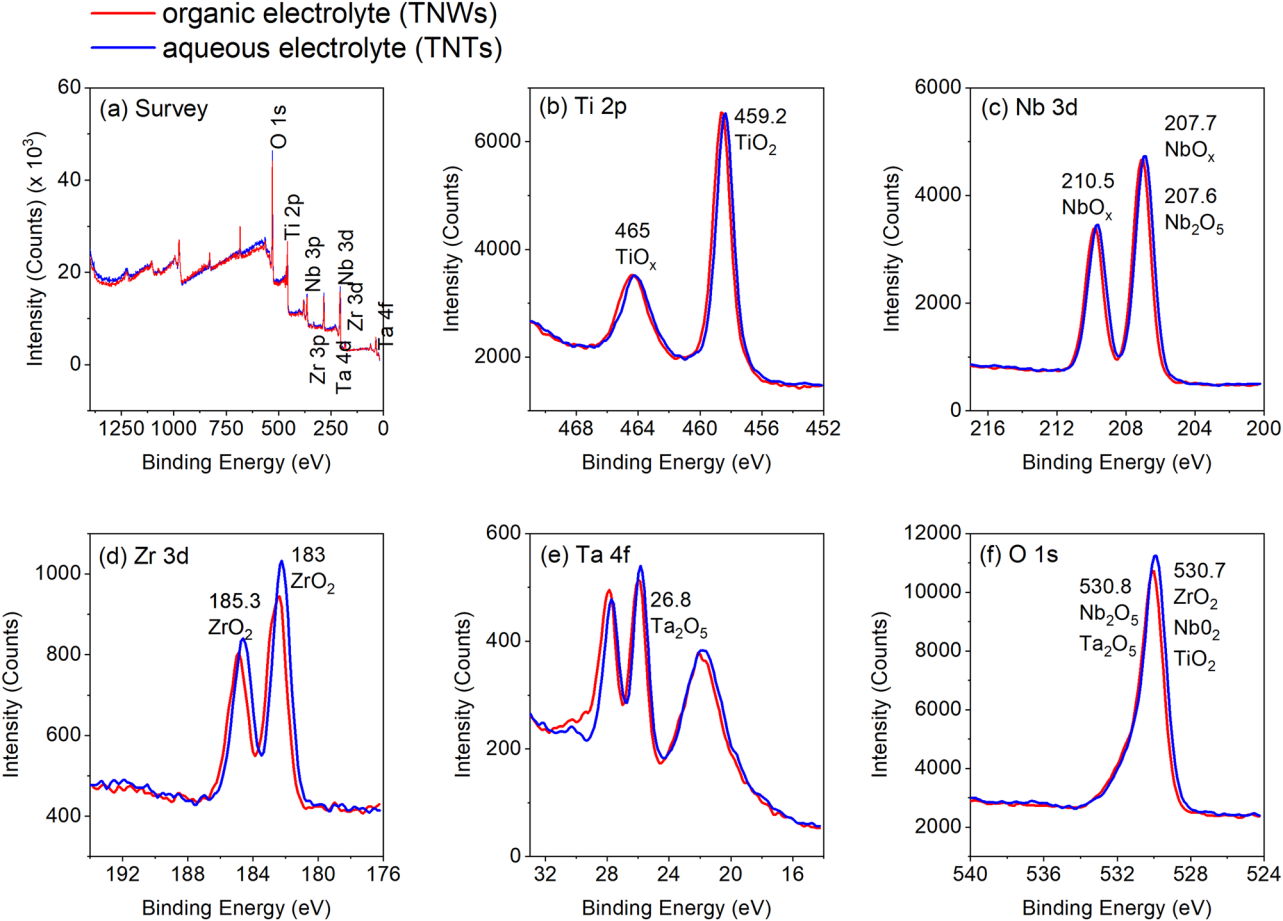
XPS analyses for the TNTs (anodization in the aqueous electrolyte) (blue line) and TNWs (anodization in the organic electrolyte) (red line). (**a**) Full survey spectrum and high-resolution spectra of (**b**) Ti 2p, (**c**) Nb 3d, (**d**) Zr 3d, (**e**) Ta 4f, and (**f**) O 1s.

Figure 4 shows the XPS analyses for the TNT and TNW samples. The survey (low resolution) spectrum (Fig. 4a) shows the presence of Ti, Nb, Zr, Ta, and O elements, as expected. The high-resolution spectra of Ti, Nb, Zr, Ta, and O (Fig. 4b–f, respectively) indicates the presence of TiO_2_, Nb_2_O_5_, Ta_2_O_5_, and ZrO_2_ oxides (NIST Standard Reference Database^25^). The binding energy curves for both samples are similar. Table 3 shows the weight percent element composition obtained from the XPS analysis. Although different electrolytes and anodization times were used for the synthesis of the TNTs and TNWs, their chemical composition is very similar, therefore it is not expected that it would influence biocompatibility.

**Table 3:** Element composition (wt. %) obtained by XPS analysis of the TNTs (anodization with the aqueous electrolyte) and TNWs (anodization with the organic electrolyte).

	O	Ti	Zr	Nb	Ta
Aqueous (TNTs)	38,9	24,1	4,8	28,5	3,8
Organic (TNWs)	38,1	25,4	4,4	27,7	4,3

**Figure 5:**
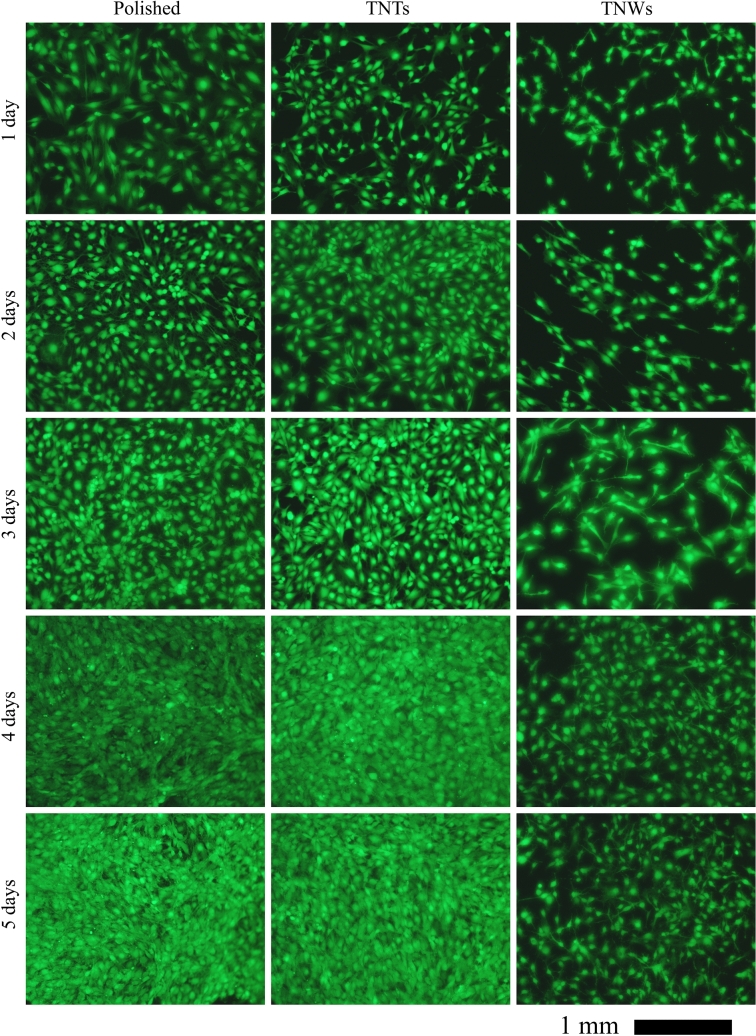
Fluorescence microscopy images of calcein-stained MC3T3-E1 cells on days 1 to 5 of culture on the surfaces of the chemically polished material, TNTs, and TNWs.

**Figure 6:**
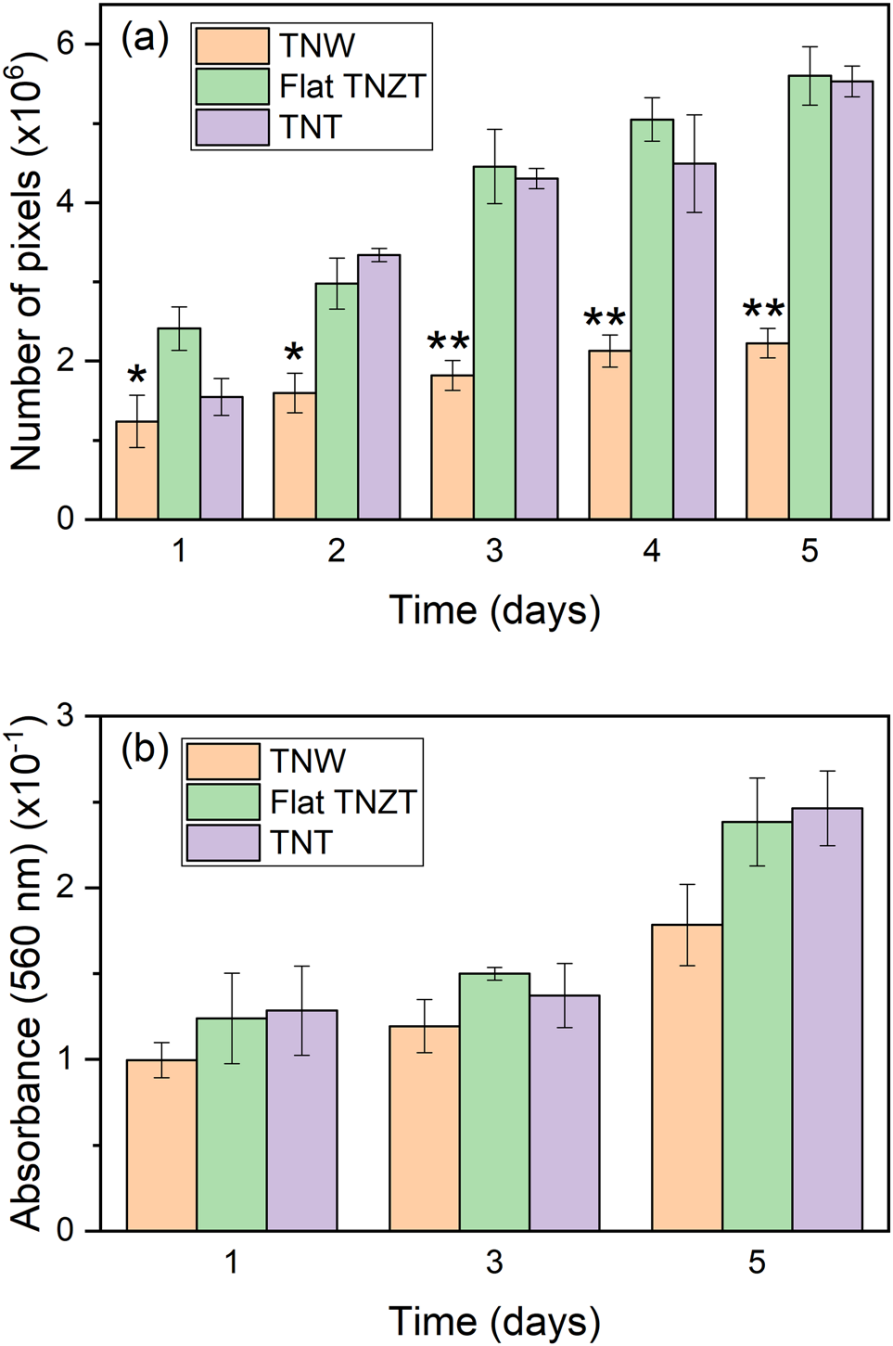
Proliferation and viability of MC3T3-E1 cells after different times of culture on the surfaces of the TNW and control samples (flat TNZT and TNT), as assessed by fluorescence microscopy (**a**) and by the MTT assay (**b**). Values represent the mean ± SEM of three independent experiments. *P ≤ 0.05, **P ≤ 0.001 vs. flat TNZT samples (two-way analysis of variance, followed by Dunnett’s post-hoc test).

The MC3T3-E1 osteoblast precursor cell line was cultured on the surfaces of TNW and control (TNT and flat TNZT) samples. Cell viability and proliferation were evaluated every 24 h for 5 days by fluorescence microscopy. Figure 5 shows a representative image of calcein-positive cells for each sample surface and each time point evaluated. An initial analysis revealed that the cells adhered to the three surfaces and proliferated. As the figure shows, the cells grown on the surfaces of the flat TNZT and TNT samples had high proliferation rates and were completely confluent after 5 days in culture. However, the cells grown on the TNW surface showed a considerably lower proliferation rate, with clearly lower numbers of cells over time than on the flat and TNT surfaces. To quantify cell proliferation, we estimated the fluorescence intensity in the microscopic images by counting the number of green pixels per image across experimental conditions (Fig. 6a). The results showed a similar number of green pixels for the flat and TNT samples, without significant differences. However, the number of pixels was significantly lower for the TNW sample at all the time points evaluated and approximately 49% lower than those for the other two samples after 5 days in culture. In addition, the viability of cells was evaluated by the MTT assay after 1, 3, and 5 days in culture (Fig. 6b). Although the differences were not statistically significant, the results showed the same general tendency as those obtained by the pixel counting method, indicating that the TNW sample has a lower number of viable cells compared to the other two surfaces after 5 days in culture.

**Figure 7:**
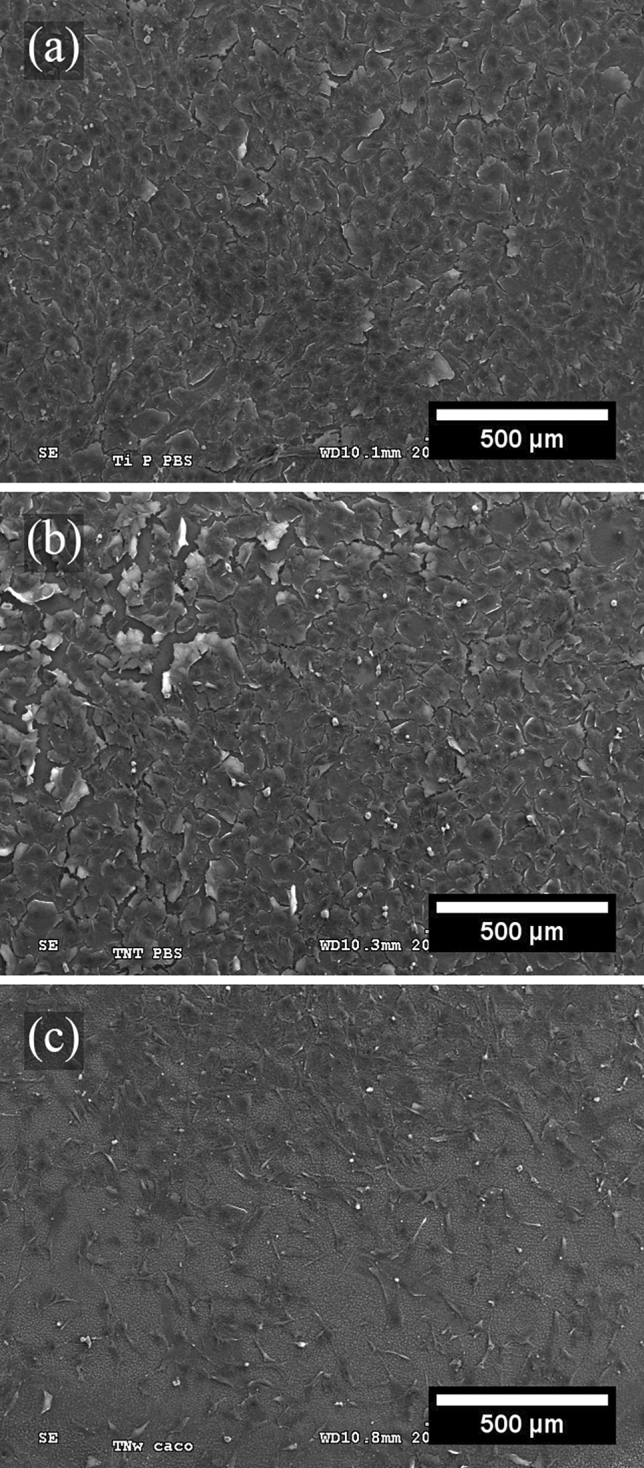
Scanning electron micrographs of MC3T3-E1 cells after 48 h of culture on the surfaces of the (**a**) flat TNZT, (**b**) TNT, and (**c**) TNW samples.

**Figure 8:**
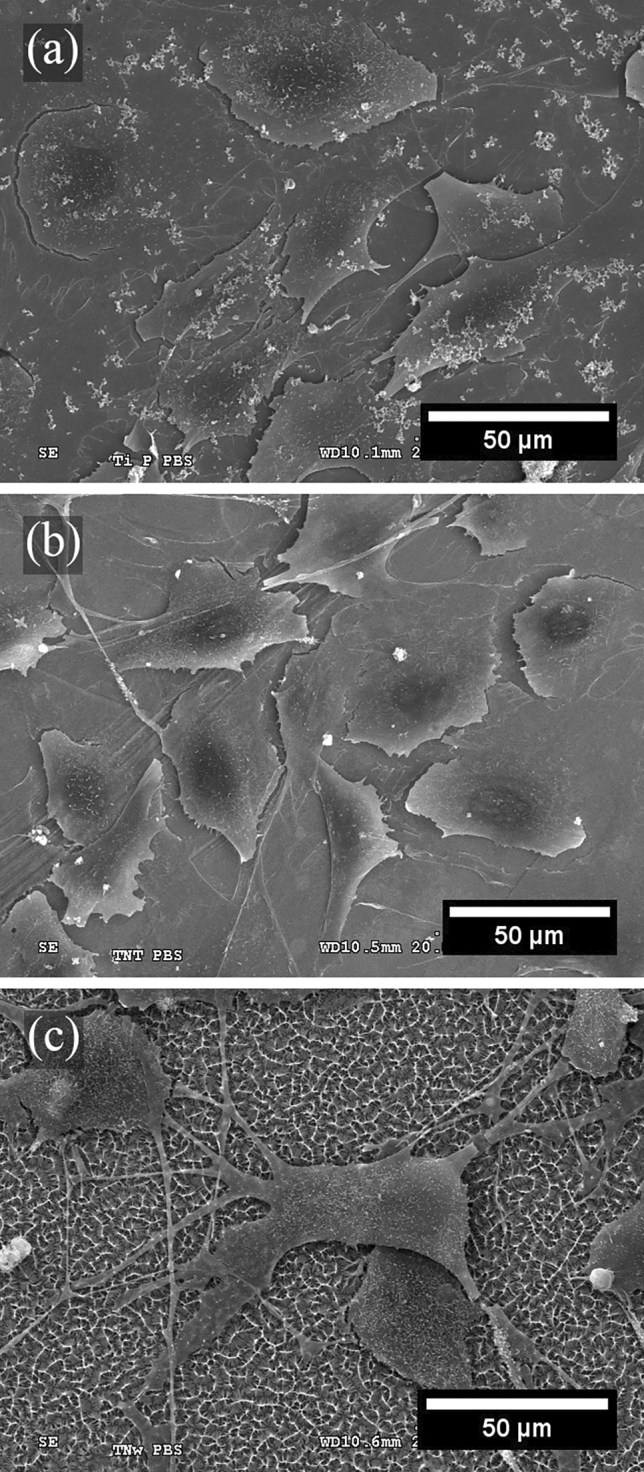
Scanning electron micrographs of MC3T3-E1 cells after 48 h of culture. (**a**) Flat TNZT, (**b**) TNTs, and (**c**) TNWs.

To observe the morphology of cells on the surfaces of the different samples in more detail, SEM was performed after 48 h of culture. Figure 7 shows low-magnification images of the cells on the TNW surface, as well as on the control samples (flat TNZT and TNT). Similar to the fluorescence microscopy images, the flat and TNT samples (Fig. 7a,b) had high densities of cells, which almost entirely covered the surfaces. By contrast, the TNW sample (Fig. 7c) showed a significantly lower number of cells. Figure 8 shows higher-magnification images of the cells on the surface of the flat TNZT, TNT, and TNW samples. Cells seeded on TNW samples seem to have a more elongated morphology and filopodia; however, future immunohistochemistry studies i.e. actin/vinculin, will help determine the impact that the different surfaces could have on cytoskeleton dynamics. Moreover, in the SEM images, TNWs can be observed on the surface, without any apparent damage that might affect cell adhesion and proliferation.

**Figure 9:**
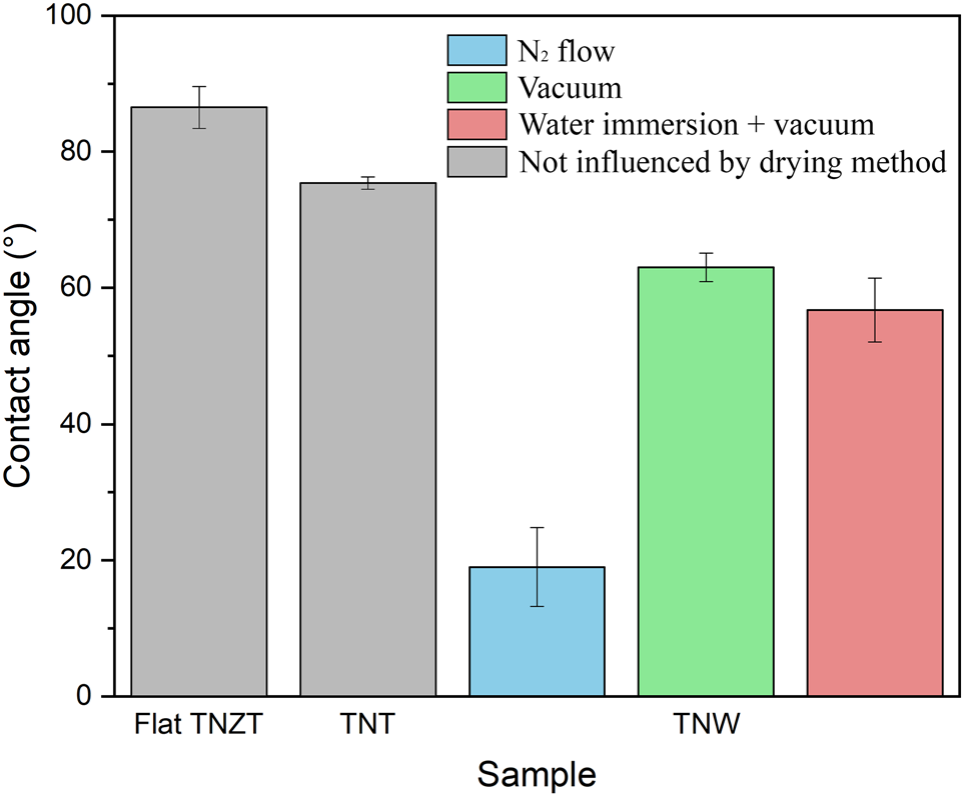
Wettability (contact angle) measurements of the flat TNZT, TNT, and TNW surfaces. The influence of the drying method after anodization for the TNW samples is shown.

One of the important properties of the biomaterials that could explain the observed differences during cell proliferation in culture is the wettability of the surface, which may affect its biocompatibility. Contact angle measurement is the most common method for assessing wettability. Figure 9 shows the results of contact angle measurements for the flat TNZT, TNT, and TNW samples (the lower the contact angle is, the higher the wettability is). The contact angle for the flat TNZT and TNT surfaces was about 86° and 75°, respectively. The wettability of the TNWs was influenced by the drying method after anodization. Drying with a N_2_ flow, which is a common method to dry anodized TNTs, resulted in a relatively low contact angle of about 19°. The TNWs obtained in this study are long and dense, which may be more difficult to dry, which raised a doubt about whether the N_2_ flow was sufficient to eliminate all water that was possibly trapped in the nanostructure. To clarify this, some TNW samples were dried under vacuum for 24 h, which resulted in a significantly higher contact angle of approximately 63°. Thus, the vacuum drying step before contact angle measurement was essential for correct measurement. In addition, some samples were immersed in water for 24 h before drying, to simulate the aqueous environment in which samples are subjected during osteoblast cell culture. This water immersion did not have a significant influence on wettability, resulting in a contact angle of about 57°.

The higher wettability of the TNTs compared with that of the flat surface was expected because of the capillary effect^10^, although the wettability may significantly vary depending on the nanotube morphology and anodization parameters^10,26,27^. To date, no study has been conducted on the wettability of anodic TNWs, but their high surface roughness and permeability could explain their significantly higher wettability. The review of Menzies and Jones^28^ about the impact of contact angle on the biocompatibility of biomaterials concluded that although a hydrophobic surface is known to reduce biocompatibility, a highly hydrophilic surface could also be detrimental because it prevents cell–cell interactions. Lee et al.^29^ studied the behavior of cells on a surface with a wettability gradient (contact angle varying from 30° to 90°) and observed that at 50° the cells presented the best adhesion. It seems that there is an intermediate contact angle (not too low or too high) which would be optimal. Although the wettability of the TNW surface seems to present a good value, other factors are probably having greater relevance to its biocompatibility.

MC3T3-E1 cells showed similar proliferation on the surfaces of the flat TNZT and TNT samples, indicating that both control samples had good biocompatibility. The high proliferation on the TNT surface was expected based on the data of other studies^17^, and similar cell proliferation on the surfaces of TNTs and flat Ti has also been observed by Chang et al.^22^. As commented in the Introduction, only a few studies have evaluated the activity of bone cells on surfaces covered with TNWs or TNFs (Table 1). Although some of these studies have shown enhanced proliferation of cells on the surfaces of TNWs compared with that on flat surfaces, reduced or similar proliferation was observed in four of the six studies listed in Table 1. The methods of synthesis as well as the morphology and dimensions of nanostructures varied widely among these studies.

Surface topography and morphology are expected to affect the TNWs biocompatibility. The nanowire/nanofiber diameter and pore size have been reported to affect the cell response. Badami et al.^30^ studied the proliferation and differentiation of MC3T3-E1 cells on polymeric fibers with diameters ranging from approximately 140 nm to 2.1 μm and observed that the smaller fiber diameters resulted in a lower cell density and prevented cell infiltration into the fibers. Infiltration is an essential parameter for bone formation because cells infiltrate and produce bone matrix proteins^16^. The diameters of TNWs/TNFs obtained by electrospinning^18,19^, thermal oxidation^20^, and atomic layer deposition^21^ were significantly larger than those of the TNWs obtained in this study or in other studies that employed electrochemical anodization as the method of synthesis. Another critical difference is that anodically grown nanowires are much denser, with practically no pores or free space.

TNWs and TNFs can be synthesized by a variety of methods, and their dimensions and morphologies may significantly vary depending on the method and parameters used. The number of studies on the proliferation of osteoblastic cells on the surface of these nanostructures is still very limited, and the results are not conclusive. Although some studies indicate that the use of TNWs/TNFs for biomedical applications is promising, the parameters affecting cell proliferation need to be better understood. Moreover, the long TNWs obtained by electrochemical anodization of the TNZT alloy provide a very high specific surface area that could be advantageous for other potential applications, such as catalysis^31^, biosensors^32^, and drug delivery systems^33^.

An additional safety concern that needs attention is the possible damage to implanted TiO_2_ nanostructures. Implants are frequently subjected to tribological conditions which could result in the release of solid debris and lead to peri-implant inflammatory reactions. As explained in “Introduction”, anodically grown TNWs are formed by the vertical split of TNTs during anodization, which results in a dual morphology formed by nanotubes with nanowires on top. This nanostructure needs to be protected from fracture or detachment from the substrate. For the TNTs, some studies can be found about their mechanical stability. Promising results were found by Shivaram et al.^34^, which performed *ex-vivo* implantation of titanium covered with TNTs and observed no significant damage for TNTs up to 1 mm long. The shear strength of TNT coatings was studied by Cao et al.^35^ and they observed that the adhesion to the substrate is higher for shorter TNTs. No study on the mechanical stability of anodic TNWs was found in the literature, a subject that will need to be addressed in the future. The diameter of anodic TNWs is considerably thinner than that of the TNWs produced by other synthesis methods (Table 2), which gives them a large flexibility (as seen by its morphology in Fig. 1) because of the lower strain for a given radius of curvature. This flexibility could possibly help to avoid mechanical damage.

## Conclusions

This study evaluated the viability and proliferation of MC3T3-E1 osteoblastic precursor cells on the surface of TNWs grown by electrochemical anodization on the TNZT alloy. TNT coated and flat TNZT were used as control samples. The TNZT alloy was confirmed to be a suitable substrate for the growth of TNWs, allowing the growth of long TiO_2_ nanostructures. MC3T3-E1 cell proliferation on the flat TNZT and TNT surfaces were similar and relatively high. Cell proliferation on the TNW sample was at least about 25% lower than that on the control samples after 5 days of culture. Despite observing that cells on the TNW sample had less metabolic activity, these differences were not statistically significant, indicating that cell survival was similar among the three different experimental conditions. The TNWs showed a moderate level of hydrophilicity, while the wettability of the control samples was considerably higher. This should, in principle, represent enhanced biocompatibility for the TNW surface; however, other factors may be playing a more important role, such as the surface topography and TNW morphology. Further studies are needed to understand all parameters affecting the proliferation of osteoblastic cells on the surface of TNWs and other similar nanostructures. The long TNWs obtained in this study have a high surface area to volume ratio that can also be useful for other applications.

## Data Availability

The datasets generated and/or analysed during the current study are available in the ZENODO repository, https://doi.org/10.5281/zenodo.6345229.
